# Space Lower Bounds for the Signal Detection Problem

**DOI:** 10.1007/s00224-020-09993-6

**Published:** 2020-07-20

**Authors:** Faith Ellen, Rati Gelashvili, Philipp Woelfel, Leqi Zhu

**Affiliations:** 1 Department of Computer Science, University of Toronto, Toronto, ON Canada; 2 Department of Computer Science, University of Calgary, Calgary, AB Canada

**Keywords:** Signal detection, ABA problem, Space complexity, Lower bounds

## Abstract

Many shared memory algorithms have to deal with the problem of determining whether the value of a shared object has changed in between two successive accesses of that object by a process when the responses from both are the same. Motivated by this problem, we define the *signal detection problem*, which can be studied on a purely combinatorial level. Consider a system with *n* + 1 processes consisting of *n* readers and one signaller. The processes communicate through a shared *blackboard* that can store a value from a domain of size *m*. Processes are scheduled by an adversary. When scheduled, a process reads the blackboard, modifies its contents arbitrarily, and, provided it is a reader, returns a Boolean value. A reader must return *true* if the signaller has taken a step since the reader’s preceding step; otherwise it must return *false*. Intuitively, in a system with *n* processes, signal detection should require at least *n* bits of shared information, i.e., *m* ≥ 2^*n*^. But a proof of this conjecture remains elusive. For the general case, we prove a lower bound of *m* ≥ *n*^2^. For restricted versions of the problem, where the processes are oblivious or where the signaller must write a fixed sequence of values, we prove a tight lower bound of *m* ≥ 2^*n*^. We also consider a version of the problem where each reader takes at most two steps. In this case, we prove that *m* = *n* + 1 blackboard values are necessary and sufficient.

## Introduction

### The Signal Detection Problem

Consider a system consisting of *n* + 1 processes, one *signaller*, *s*, and *n* *readers*, $r_{1},\dots ,r_{n}$, that communicate through a shared blackboard. The blackboard can contain one value from a domain of size *m*. Processes are scheduled to take steps one at a time by an adversarial scheduler. Whenever a process takes a step, it atomically reads the blackboard and can modify its contents arbitrarily without interruption from other processes.

In the *signal detection problem*, each time a reader, *r*_*i*_, has taken a step, it must return a Boolean value. If *r*_*i*_ has no preceding step, it can return either *true* or *false*. Otherwise, it must return *true* if and only if the signaller has taken a step since *r*_*i*_’s preceding step. We are concerned with how large *m* has to be for this problem to be solvable.

### Simple Signal Detection Algorithms

For large or unbounded values of *m*, there are some simple solutions to the signal detection problem. For example, the blackboard could store an unbounded *signal counter* that is initially 0. Each time the signaller takes a step, it increments the counter. When a reader is scheduled, it simply memorizes the counter value, but does not change it. To detect whether a signal has occurred since its last step, a reader only needs to compare the current counter value with the one it read in its previous step. The value stored on the blackboard grows with the number of signals that have occurred, which can be unbounded.

A similar algorithm is for the signaller to increment the counter whenever it sees the value is odd and for each reader to increment the counter whenever it sees the value is even. A reader also memorizes the resulting value of the counter. It detects whether a signal has occurred since its last step by comparing this value with the value it previously memorized. If there are many consecutive steps by the signaller, the value of the counter grows more slowly than in the previous algorithm.

The following simple algorithm stores an *n*-bit string $(b_{1},\dots ,b_{n})$ on the blackboard, i.e. the domain size is *m* = 2^*n*^. Initially, $b_{1}=\dots =b_{n}=0$. Whenever the signaller takes a step, it sets all bits to 1. For each $j\in \{1,\dots ,n\}$, reader *r*_*j*_ resets bit *b*_*j*_ to 0, it returns *false* if the old value of *b*_*j*_ was 0, and it returns *true* if the old value of *b*_*j*_ was 1.

### ABA Detection

Signal detection is related to the fundamental *ABA detection* problem in asynchronous shared memory systems. In such systems, a process that observes the same value *A* in some shared object in two successive accesses cannot tell whether the value of the object remained unchanged between them. More formally, it cannot distinguish between an execution in which the shared object did not change and an execution in which the value of the object changed from *A* to some other value *B* and then back to *A*. Many shared memory algorithms have to deal with this problem.

A well-known example is the double-collect algorithm for performing an atomic *scan* of an array [1]: A process repeatedly performs a *collect* (reading all components of the array one by one) until the sequences of values read in two consecutive collects are the same. This algorithm is only correct (linearizable) if no ABAs occur, meaning that any two consecutive reads of the same array entry return the same value if and only if the value of the array entry was not changed between the two reads. This is because it can be shown that, provided no ABAs occur, the sequence returned by a scan must be the contents of the array at the end of its second last collect and the beginning of its last collect. However, in executions in which ABAs occur, this implementation might incorrectly return a sequence of values that was not the contents of the array at any point during the execution.

A standard approach to dealing with ABAs is tagging, as introduced by IBM [4], whereby a shared object gets augmented with a tag that changes with every write operation. If tags are never reused, the ABA problem can be avoided. From a theory perspective, this solution is unsatisfactory: If there is no bound on the length of executions, then unbounded sized objects are required to accommodate ever increasing tag values. Even though, in many practical scenarios, a system may never run out of tags, it is often desirable or even necessary to use an entire word for data. In such scenarios, the tag associated with a data word could be stored in a subsequent memory location and double-width atomic instructions could be used. However, these are not supported by most of today’s mainstream architectures [7].

In some cases, it is possible to store the tag in an unrelated memory location [6], but this requires technically difficult algorithms and tedious correctness proofs. As a result, algorithm designers often deal with ABAs in an ad-hoc way. For example, a pair of handshaking bits between each pair of processes can be used to detect changes in the components of the array in a wait-free implementation of a snapshot object [1]. Such solutions are algorithm specific and require individual correctness proofs.

ABAs can also occur when using compare-and-swap (CAS) objects, which are provided by most existing multiprocessor systems and are much more powerful than read/write registers. Algorithms devised in theoretical research often use load-linked store-conditional (LL/SC) objects, which do not suffer from ABAs, and can easily replace CAS objects. Unfortunately, only a small number of multiprocessor systems provide LL/SC and they are weaker than the LL/SC specification used in theoretical research. Variants of LL/SC available in modern hardware restrict programmers severely in how the objects can be used [9], and “offer little or no help with preventing the ABA problem” [8].

To study the complexity of ABA detection, Aghazadeh and Woelfel [2] defined an *ABA detecting register*, which extends a read/write register with the ability to detect ABAs. It supports the operations DWrite(*x*), which changes the value of the object to *x*, and DRead(), which returns the current value of the object together with a Boolean flag. The flag is true if and only if the process has previously performed DRead() and, since its last preceding DRead(), some process performed DWrite(). The authors proved space lower bounds and time-space-tradeoffs for linearizable implementations of ABA detecting registers in shared memory systems with *n* processors that provide bounded atomic base objects, such as bounded read/write registers or bounded CAS objects. For example, if only bounded read/write registers are available as base objects, then at least *n* − 1 of them are needed to obtain an obstruction-free ABA detecting register. If bounded CAS objects are also available, then any implementation using *m* base objects has step-complexity Ω(*n*/*m*).

All the lower bound results in [2] are specific to the base objects provided by the system, and provide no insights for systems using different sets of base objects. But we conjecture that there is a large, general lower bound for the amount of information that needs to be stored in a system for processes to detect ABAs: Intuitively, the system state needs to keep track of whether the value of the object has changed since each process last accessed the object. This requires at least *n* bits of information. Hence, it seems believable that detecting ABAs in any system with arbitrarily powerful base objects requires at least *n* bits of information to be stored either in the base object or in the hardware implementing the base objects (for example, implementing LL/SC objects). Using the reasonable assumption that a single base object can store $O(\log n)$ bits of information, this would imply that ${\Omega }(n/\log n)$ base objects are required for implementing a single ABA detecting object.

The signal detection problem is a restricted version of the problem of detecting ABAs in asynchronous shared memory systems, stripped down to the essentials necessary for determining the information theoretic requirements. Its definition is self-contained, and the problem can be studied without any background knowledge of shared memory systems. If *n* processes can detect ABAs in a standard asynchronous shared memory system with arbitrarily strong primitives, then they can also solve signal detection. A reader simply remembers the last value it read from the blackboard. When it reads the blackboard again, it returns *true* if it sees a different value or it detected an ABA; otherwise it returns *false*. Therefore, if signal detection requires that the blackboard has domain size at least *m*^∗^, then $\log _{2} m^{\ast }$ is a lower bound for the number of bits needed for ABA detection.

### Results

We conjecture that any solution to the signal detection problem requires the domain size, *m*, of the blackboard to be at least 2^*n*^. Although we prove this conjecture when there are *n* ≤ 2 readers, a proof when *n* ≥ 3 has eluded us. This simply defined combinatorial problem does not seem to have a simple solution. Even a proof of a polynomial lower bound is surprisingly non-trivial. We show the following result in Section 6.

#### **Theorem 1**

In any algorithm for the signal detection problem, the blackboard has domain size at least *n*(*n* + 1)/2.

To obtain better understanding, we consider several restricted versions of the signal detection problem and prove tight upper and lower bounds for them.

First, we consider the *b*-read-bounded version of signal detection, where no reader takes more than *b* steps, but the signaller can take arbitrarily many steps. In this case, the second algorithm from Section 1.2 uses a domain of size 2*bn* + 1. We show how to improve this algorithm.

#### **Theorem 2**

For *b* ≥ 2, the *b*-read-bounded signal detection problem can be solved using a blackboard with domain size (*b* − 1)*n* + 1.

Thus, the *b*-read-bounded problem is strictly easier than the unrestricted problem when *b* ≤⌈*n*/2⌉. For *b* = 2, we also prove that this algorithm is optimal.

#### **Theorem 3**

In any algorithm for the 2-read-bounded signal detection problem, the blackboard has domain size at least *n* + 1.

Next, we consider signal detection when the actions of the signaller do not depend on what steps the readers have taken. Signal detection with *fixed signals* is the special case where the signaller writes the same sequence of values to the blackboard in every execution. Note that the simple algorithm above with *m* = 2^*n*^ uses fixed signals.

#### **Theorem 4**

In any algorithm for the signal detection problem with *fixed signals*, the blackboard has domain size at least 2^*n*^.

Then we consider the case of *write oblivious* processes. Here each process *p* is equipped with a fixed function $f_{p}:\{0,\dots ,m-1\}\to \{0,\dots ,m-1\}$. When taking a step it replaces the blackboard contents *x* with *f*_*p*_(*x*). Hence, what a process writes to the blackboard is independent of the internal state of the process. However, the return value of a reader’s step may depend on its internal state. In the simple algorithm above, which uses *m* = 2^*n*^ blackboard values, processes are write oblivious. We prove that, when processes are write oblivious, no better algorithm exists.

#### **Theorem 5**

In any algorithm for the signal detection problem with write oblivious processes, the blackboard has domain size at least 2^*n*^.

The signal detection problem with write oblivious processes is similar to determining the minimum size of a dictionary in a sequential system. A *dictionary* supports three operations, *insert*(*x*), *query*(*x*), and *reset*(), where *x* is a parameter chosen from a domain of size *n*. A call to *query*(*x*) returns true if there has been an *insert*(*x*) operation since the last *reset*() operation or since the beginning of the execution, if there has been no *reset*(). Otherwise, it returns false. A dictionary implemented using *b*(*n*) bits immediately yields a solution to the signal detection problem with oblivious processes as follows: A blackboard with *m* = 2^*b*(*n*)^ possible values is used to store the dictionary. When a signaller takes a step, it simulates a *reset*() operation on the dictionary stored on the blackboard. Similarly, when reader *r*_*i*_ takes a step, it simulates *query*(*i*) followed by *insert*(*i*) on the dictionary and then returns the return value of its query operation. However, an arbitrary solution to the signal detection problem does not seem to yield an implementation of a dictionary. The difficulty is that the return value of a step by a reader *r*_*i*_ can depend on the state of the reader and, thus, its entire past execution. In contrast, the result of a *query*(*i*) operation is only a function of the state of the dictionary. Hence, the *n*-bit information theory lower bound for implementing a dictionary cannot be used to obtain Theorem 5.

In the simple algorithm that uses *m* = 2^*n*^ blackboard values, the response each reader returns also does not depend on its internal state, but only on the contents of the blackboard. We call such processes *response oblivious*. The same lower bound holds for any algorithm with response oblivious processes, even if the algorithm supports only 2 steps by each reader.

#### **Theorem 6**

In any algorithm for 2-read-bounded signal detection with response oblivious processes, the blackboard has domain size at least 2^*n*^.

Theorem 2 implies that, for the unrestricted signal detection problem when there is only *n* = 1 reader, the domain size is at least 2 = 2^*n*^. In Section 7, we investigate the unrestricted signal detection problem for the case of *n* = 2 readers. First, we prove a tight lower bound of 4 for the size of the blackboard domain. Then we present an algorithm for two readers, *r*_1_ and *r*_2_, which uses a bounded number of blackboard values, and for which every reachable configuration *C* satisfies the following property: As long as only readers take steps starting in *C*, at most three different blackboard values are obtained. This observation indicates that a tight lower bound of *m* ≥ 2^*n*^ for *n* readers for the unrestricted signal detection problem may have to be fundamentally different from our lower bound proof for fixed signals. In that proof, we showed that one can reach a configuration, *C*, from which 2^*n*^ different blackboard values result from the 2^*n*^ schedules that are sub-sequences of $(r_{1},\dots ,r_{n})$. In our third simple algorithm for *n* readers in Section 1.2, each execution that ends with the signaller taking a step results in a configuration with this property. But our two reader algorithm indicates that a lower bound proof for the unrestricted signal detection problem cannot rely on this property.

## Preliminaries

We consider a deterministic, asynchronous system in which *n* + 1 processes, $s,r_{1},\dots ,r_{n}$ communicate with one another using a single shared *blackboard*. Each time a process takes a *step*, it atomically reads the blackboard, may change the value of the blackboard based on its state and the value it read, and updates its state.

A *configuration* *C* consists of a value, *v*(*C*), for the blackboard and a state for each process. An *execution* is an alternating sequence of configurations and steps, starting and ending with a configuration, such that each step can be performed in the configuration that precedes it, resulting in the configuration that follows it. Configuration *C* is *reachable* if there is an execution starting with an initial configuration and ending with *C*. For any set of processes, *P*, a *P*-*only execution* is an execution in which only processes in *P* take steps in the execution. A solo execution is a *P*-only execution in which *P* contains only one process, i.e., all steps in the execution are by the same process.

A *schedule* is a sequence of processes (in which the same process can occur multiple times). A *P*-*only schedule* is a schedule in which only processes in *P* appear. For any deterministic algorithm and for any configuration *C*, a schedule determines a unique execution starting from *C* in which the processes take steps in the order specified by the schedule. If *α* is a finite schedule, then *Cα* denotes the configuration at the end of this execution.

Two configurations, *C* and $C^{\prime }$, are *indistinguishable* to a set of processes, *P*, if $\mathsf {v}(C) = \mathsf {v}(C^{\prime })$ and each process in *P* has the same state in *C* as it does in $C^{\prime }$. If *C* and $C^{\prime }$ are indistinguishable to *P* and *α* is a finite *P*-only schedule, then the same sequence of steps is taken in the executions determined by *α* from *C* and $C^{\prime }$. Moreover, *Cα* and $C^{\prime }\alpha $ are indistinguishable to *P*.

Given a set of readers, *R*, let **R** denote the schedule consisting of one occurrence of each reader in *R*, in order of their identifiers, and let *M*(*R*) denote the set {*r*_*i*_ : *i* ≤ *j* for some *r*_*j*_ ∈ *R*} of all readers whose identities are less than or equal to the largest identity of the readers in *R*. In particular, *M*(*∅*) = *∅*. For example, $M(\{r_{1},r_{4},r_{8}\}) = \{r_{1},r_{2},\dots ,r_{8}\}$. Notice that, for any two sets of readers *R* and $R^{\prime }$, either $M(R) \subseteq M(R^{\prime })$ or $M(R^{\prime }) \subseteq M(R)$. There are *n* + 1 such sets, i.e., $|\{M(R) : R \subseteq \{r_{1},\dots ,r_{n}\}\}| = n+1$.

### **Lemma 1**

For every signal detection algorithm in which the domain size of the blackboard is finite, there is a reachable configuration *D* such that, for every set of readers, *T*, *v*(*D***T***s*β_*T*_) = *v*(*D***T**) for some (*M*(*T*) ∪{*s*})-only schedule β_*T*_.

### *Proof*

Consider any signal detection algorithm. Assume that, for each reachable configuration *C*, there is a set of readers, *T*, such that *v*(*C***T***s*β)≠*v*(*C***T**) for all (*M*(*T*) ∪{*s*})-only schedules β. We define an infinite sequence of reachable configurations as follows. Let *C*_0_ be the initial configuration. Let *j* ≥ 1 and suppose that *C*_*j*− 1_ is a reachable configuration. Let *T*_*j*_ be a set of readers such that *v*(*C*_*j*− 1_**T**_*j*_*s*β)≠*v*(*C*_*j*− 1_**T**_*j*_) for all (*M*(*T*_*j*_) ∪{*s*})-only schedules β. The existence of *T*_*j*_ follows from the assumption, since *C*_*j*− 1_ is reachable. Let *C*_*j*_ = *C*_*j*− 1_**T**_*j*_*s*.

Since there is only a finite number of readers, there exists a set *M* such that $\{ j \in \mathbb {Z}^{+} : M(T_{j}) = M\}$ is infinite. Let *M* be the largest such set, let $J = \{ j \in \mathbb {Z}^{+} : M(T_{j}) = M\}$, and let $k^{*} = {\min \limits } \{ k \geq 1 : M(T_{j}) \subseteq M \textrm { for all } j \geq k \}$. Note that, for all *k*,*ℓ* ∈ *J* such that *k*^∗^≤ *k* < *ℓ*, the schedule **T**_*k*+ 1_*s***T**_*k*+ 2_*s*⋯**T**_*ℓ*_ is (*M* ∪{*s*})-only. Thus, by definition of *T*_*k*_, *v*(*C*_*k*_**T**_*k*_)≠*v*(*C*_*k*_**T**_*k*_*s***T**_*k*+ 1_*s*⋯**T**_*ℓ*_) = *v*(*C*_*ℓ*_**T**_*ℓ*_). Hence, the domain size of the blackboard is infinite. □

## Read-Bounded Signal Detection

In the *b*-read-bounded signal detection problem, no reader takes more than *b* steps, but the signaller can take arbitrarily many steps.

Consider the following algorithm that solves this restricted problem for *b* = 2 using a blackboard with domain size *m* = *n* + 1:

- The blackboard initially has value 0.
- Whenever *s* takes a step, it resets the blackboard contents to 0.
- When *r*_*i*_ takes its first step, it changes the blackboard contents to *i* if it reads 0; otherwise it leaves the blackboard unchanged. In either case, *r*_*i*_ locally stores the value *v*_*i*_≠ 0 of the blackboard immediately after its first step and returns *true*.
- When *r*_*i*_ takes its second step, it returns *false* if it reads *v*_*i*_ from the blackboard; otherwise it returns *true*. It does not change the value of the blackboard in either case.

Note that only the signaller changes the blackboard contents to 0 and readers only change the blackboard contents from 0. Thus, if the signaller does not take any steps between the two steps of reader *r*_*i*_, then the value of the blackboard remains *v*_*i*_ during this interval and *r*_*i*_ returns *false*.

If the signaller does take a step between the two steps of reader *r*_*i*_, then the blackboard is reset to 0. Consider the last step, $S^{\prime }$, by the signaller during this interval. If no reader takes its first step after $S^{\prime }$, but before the second step by *r*_*i*_, then *r*_*i*_ will read 0 from the blackboard on its second step and return *true*. Otherwise, consider the first step after $S^{\prime }$ in which a reader *r*_*j*_ takes its first step. It will change the blackboard contents to *j*. Note that *j*≠*v*_*i*_, since *r*_*j*_ is the only reader that can change the blackboard contents to *j* and *r*_*j*_ has not previously taken a step. In this case, *r*_*i*_ will read *j* from the blackboard on its second step and return *true*.

A similar algorithm works for *b*-read-bounded signal detection for any larger *b*, but the size of the blackboard domain also increases.

### **Theorem 2**

For *b* ≥ 2, the b-read-bounded signal detection problem can be solved using a blackboard with domain size (*b* − 1)*n* + 1.

### *Proof*

Consider the following algorithm for a signaller and *n* readers:

- The blackboard domain is {0}∪{(*i*,*j*) : 1 ≤ *i* ≤ *n* and 1 ≤ *j* ≤ *b* − 1}. The blackboard initially has value 0.
- Whenever *s* takes a step, it resets the blackboard contents to 0.
- Each reader *r*_*i*_ has a local *b*-bounded counter *c*_*i*_, which is initially 0 and is incremented each time *r*_*i*_ reads 0 from the blackboard. It also has a local variable *v*_*i*_ in which it stores a non-zero value from the blackboard domain. It initially contains (*i*,1).
- When *r*_*i*_ takes a step, it reads the blackboard. It will return *false* if the value read is the same as the value stored in *v*_*i*_. Otherwise, it returns *true*. If the value read is non-zero, it stores the result in *v*_*i*_ and does not change the blackboard. If *r*_*i*_ reads 0, it increments *c*_*i*_ and, if *c*_*i*_ < *b*, it changes the blackboard to (*i*,*c*_*i*_) and also sets *v*_*i*_ to the same value. When *c*_*i*_ = *b*, reader *r*_*i*_ can take no additional steps.

The counter *c*_*i*_ is initially 0. Before changing the blackboard to (*i*,*c*_*i*_), reader *r*_*i*_ increments *c*_*i*_ and checks that it is less than *b*. Hence, the blackboard only contains values in its domain. Note that only *r*_*i*_ changes the blackboard to (*i*,*j*) for 1 ≤ *j* ≤ *b* − 1. Thus, whenever the blackboard is changed to a nonzero value, its new value is a value that it never previously had.

Since the blackboard initially contains 0 and *v*_*i*_ initially contains (*i*,1), the first time *r*_*i*_ takes a step, it will read a value different from *v*_*i*_ and will return *true*.

At the end of each step by *r*_*i*_, the blackboard contains *v*_*i*_. Readers only change the blackboard when it contains 0. Thus, if the signaller does not take any steps between two steps of reader *r*_*i*_, then the value of the blackboard remains *v*_*i*_ during this interval and *r*_*i*_ returns *false*.

If the signaller does take a step between two consecutive steps of reader *r*_*i*_, then the blackboard is reset to 0. Then either the blackboard has value 0 when it is later read during the second of these steps, or some other process changed the blackboard to have a value it never previously had. In either case, its value is not equal to *v*_*i*_ and *r*_*i*_ returns *true*.

The counter *c*_*i*_ is incremented each time *r*_*i*_ reads 0 from the blackboard. Since it is initially 0 and otherwise is not incremented, it records the number of times that *r*_*i*_ reads 0 from the blackboard. If *r*_*i*_ takes at most *b* steps, then *c*_*i*_ < *b* at the beginning of each of its steps, so *r*_*i*_ does not stop prematurely. □

Note that, if *b* ≤⌈*n*/2⌉, then (*b* − 1)*n* + 1 ≤ (*n* − 1)*n*/2 + 1 < *n*(*n* + 1)/2 for *n* ≥ 2. Hence, the domain size used in this algorithm is strictly less than the lower bound in Theorem 1 for any algorithm solving signal detection with an unbounded number of reads.

When *b* = 2, we can show that the domain size of this algorithm cannot be any smaller. The following simple lemma is the key to our lower bound for 2-read-bounded signal detection.

### **Lemma 2**

Let *C* be a configuration and let *r* be a reader. If *α* is an $(\{r_{1},\dots ,r_{n}\} - \{r\})$-only schedule and β is a $(\{s,r_{1},\dots ,r_{n}\}-\{r\})$-only schedule, then, for every configuration *D* in the execution determined by *α* from $C^{\prime } = Cr$ and for every configuration *E* in the execution determined by β from $C^{\prime }\alpha s$, *v*(*D*)≠*v*(*E*).

### *Proof*

Suppose not. Then there are some such configurations *D* and *E* such that *v*(*D*) = *v*(*E*). Since *r* does not occur in the schedule *αs*β, *D* and *E* are indistinguishable to *r*. Note that *r* must return *false* if it takes a step in configuration *D*, because *s* has not taken any steps since *r* last took a step. However, *r* must return *true* if it takes a step in configuration *E*, because *s* has taken a step since *r* last took a step. This is impossible, because *D* and *E* are indistinguishable to *r*. □

We can now prove Theorem 3.

### **Theorem 3**

In any algorithm for the 2-read-bounded signal detection problem, the blackboard has domain size at least *n* + 1.

### *Proof*

Let *C*_0_ be the initial configuration. For 1 ≤ *j* ≤ *n*, let *C*_*j*_ = *C*_*j*− 1_*sr*_*j*_ and let *C*_*n*+ 1_ = *C*_*n*_*s*. Let *α* denote the empty schedule.

For 1 ≤ *i* < *n*, let β denote the schedule *r*_*i*+ 1_⋯*sr*_*n*_*s*. By Lemma 2 with $C^{\prime } = C_{i}$ and *r* = *r*_*i*_, *v*(*C*_*i*_)≠*v*(*E*) for all configurations *E* in the execution starting from *C*_*i*_*s* determined by β. In particular, *v*(*C*_*i*_)≠*v*(*C*_*j*_) for *i* + 1 ≤ *j* ≤ *n* + 1.

For *i* = *n*, let β denote the empty schedule. By Lemma 2 with $C^{\prime } = C_{n}$ and *r* = *r*_*n*_, *v*(*C*_*n*_)≠*v*(*C*_*n*+ 1_).

Hence $|\{\mathsf {v}(C_{1}),\dots ,\mathsf {v}(C_{n}), \mathsf {v}(C_{n+1})\}| = n+1$. □

## Fixed Signals

Suppose that, whenever the signaller takes a step, the resulting value of the blackboard does not depend on what its value was. In other words, for all *k* ≥ 1, there exists a value *v*_*k*_ in the blackboard domain such that, immediately after the signaller’s *k*’th step in any execution, the blackboard has value *v*_*k*_. For example, this is the case if the signaller always resets the contents of the blackboard to a fixed value, say 0. Using Lemma 1, we can show that, under this restriction, the blackboard has domain size at least 2^*n*^.

### **Theorem 4**

In any algorithm for the signal detection problem with fixed signals, the blackboard has domain size at least 2^*n*^.

### *Proof*

Suppose the domain size of the blackboard is finite. Then, by Lemma 1, it is possible to reach a configuration *D* such that, for any set of readers *T*, there is a (*M*(*T*) ∪{*s*})-only schedule β_*T*_ such that *v*(*D***T***s*β_*T*_) = *v*(*D***T**).

Suppose there exist two different sets of readers $R, R^{\prime } \subseteq \{r_{1},\dots ,r_{n}\}$ such that $\mathsf {v}(D\mathbf {R}) = \mathsf {v}(D\mathbf {R^{\prime }})$. Without loss of generality, **R** = **T***x***X** and $\mathbf {R^{\prime }} = \mathbf {T} \mathbf {X^{\prime }}$, where $x \in R - R^{\prime }$ and **T** is the longest common prefix of **R** and $\mathbf {R}^{\prime }$. Note that *M*(*T*) is disjoint from $\{x\} \cup X \cup X^{\prime }$, since **R** and $\mathbf {R}^{\prime }$ are sorted. By definition of *D*, there is a (*M*(*T*) ∪{*s*})-only schedule β_*T*_ such that *v*(*D***T***s*β_*T*_) = *v*(*D***T**). The signaller *s* occurs exactly once in each of the schedules **T***s* and **T***xs*. Since *s* has fixed signals, *v*(*D***T***s*) = *v*(*D***T***xs*). The configurations *D***T***s* and *D***T***xs* are indistinguishable to *M*(*T*), so, by induction on the length of β, the configurations *D***T***s*β and *D***T***xs*β are indistinguishable to *M*(*T*) for every prefix β of β_*T*_. In particular, *v*(*D***T***xs*β_*T*_)= *v*(*D***T***s*β_*T*_) = *v*(*D***T**). Since *M*(*T*) ∪{*s*,*x*} is disjoint from $X^{\prime }$, configurations *D***T***xs*β_*T*_ and *D***T** are indistinguishable to the set of readers $X^{\prime }$. Thus $\mathsf {v}(D\mathbf {T}xs{\upbeta }_{T}\mathbf {X^{\prime }}) = \mathsf {v}(D\mathbf {T}\mathbf {X^{\prime }}) = \mathsf {v}(D\mathbf {R^{\prime }}) = \mathsf {v}(D\mathbf {R}) = \mathsf {v}(D\mathbf {T}x\mathbf {X})$. Since $ x \notin M(T) \cup X \cup X^{\prime } \cup \{s\}$, it follows that $D\mathbf {T}xs{\upbeta }_{T}\mathbf {X^{\prime }}$ and *D***T***x***X** are indistinguishable to *x*. Note that *x* must return *false* if it takes a step in configuration *D***T***x***X**, because *s* has not taken any steps since *x* last took a step. However, *x* must return *true* if it takes a step in configuration $D\mathbf {T}xs{\upbeta }_{T}\mathbf {X^{\prime }}$, because *s* has taken a step since *x* last took a step. This is impossible, because these two configurations are indistinguishable to *x*.

Hence, $\mathsf {v}(D\mathbf {R}) \neq \mathsf {v}(D\mathbf {R^{\prime }})$ for all different sets of readers *R* and $R^{\prime }$. It follows that $|\{\mathsf {v}(D\mathbf {R}) : R \subseteq \{r_{1},\dots ,r_{n}\}\}| = 2^{n}$. □

## Oblivious Processes

In this section, we consider processes whose actions are oblivious to their states, either when writing to the blackboard or responding to a read.

Recall that a process is write oblivious if what it writes to the blackboard in a step only depends on the value of the blackboard at the beginning of that step. We prove that the simple algorithm in Section 1.2 with domain size 2^*n*^ is optimal if both the signaller and the readers are write oblivious.

### **Theorem 5**

In any algorithm for the signal detection problem with write oblivious processes, the blackboard has domain size at least 2^*n*^.

### *Proof*

Suppose the domain size of the blackboard is finite. For any (possibly empty) set of readers *R* and any positive integer *i*, consider the schedule (*s***R**)^*i*^ which consists of *s***R** repeated *i* times. Because the domain size of the blackboard is finite, there exist 0 < *i* < *j* such that *v*(*C*_0_(*s***R**)^*i*^) = *v*(*C*_0_(*s***R**)^*j*^), where *C*_0_ is the initial configuration.

Let $R^{\prime } \neq R$ be a different set of readers and let $0 < i^{\prime } < j^{\prime }$ be such that $\mathsf {v}(C_{0}(s\mathbf {R^{\prime }})^{i^{\prime }}) = \mathsf {v}(C_{0} (s\mathbf {R^{\prime }})^{j^{\prime }})$. Without loss of generality, suppose there is a reader $r_{k} \in R^{\prime } - R$.

To obtain a contradiction, assume that $\mathsf {v}(C_{0}(s\mathbf {R})^{i}) = \mathsf {v}(C_{0}(s\mathbf {R^{\prime }})^{i^{\prime }})$. Since processes are write oblivious, it follows that $\mathsf {v}(C_{0} (s\mathbf {R^{\prime }})^{i^{\prime }}(s\mathbf {R})^{j-i}) = \mathsf {v}(C_{0} (s\mathbf {R})^{i} (s\mathbf {R})^{j-i}) = \mathsf {v}(C_{0} (s\mathbf {R})^{j}) = \mathsf {v}(C_{0} (s\mathbf {R})^{i}) = \mathsf {v}(C_{0}(s\mathbf {R^{\prime }})^{i^{\prime }})$. Since *r*_*k*_ takes no steps in (*s***R**)^*j*−*i*^, configurations $C_{0} (s\mathbf {R^{\prime }})^{i^{\prime }}(s\mathbf {R})^{j-i}$ and $C_{0}(s\mathbf {R^{\prime }})^{i^{\prime }}$ are indistinguishable to *r*_*k*_. The signaller has taken a step after *r*_*k*_’s last step in the execution determined by $(s\mathbf {R^{\prime }})^{i^{\prime }}(s\mathbf {R})^{j-i}$ starting from *C*_0_, so *r*_*k*_ must return *true* if it takes a step in configuration $C_{0} (s\mathbf {R^{\prime }})^{i^{\prime }}(s\mathbf {R})^{j-i}$. However, the signaller has not taken a step after *r*_*k*_’s last step in the execution determined by $(s\mathbf {R^{\prime }})^{i^{\prime }}$ starting from *C*_0_, so *r*_*k*_ must return *false* if it takes a step in configuration $C_{0}(s\mathbf {R^{\prime }})^{i^{\prime }}$. This is impossible, so $\mathsf {v}(C_{0}(s\mathbf {R})^{i}) \neq \mathsf {v}(C_{0}(s\mathbf {R^{\prime }})^{i^{\prime }})$.

Since this holds for any two different sets of readers *R* and $R^{\prime }$, it follows that the blackboard domain has size at least 2^*n*^. □

We now consider response oblivious readers, whose responses only depend on the contents of the blackboard, but not their current state. However, processes can modify the blackboard based on both the blackboard contents and their current state. Even for the 2-read-bounded signal detection problem, at least 2^*n*^ different blackboard values are necessary when readers are response oblivious.

### **Theorem 6**

In any algorithm for 2-read-bounded signal detection with response oblivious processes, the blackboard has domain size at least 2^*n*^.

### *Proof*

Consider a set of readers *R* and let *Q* = {*r*_1_,…,*r*_*n*_}− *R*. Note that, in the execution from the initial configuration *C*_0_ determined by schedule **Q***s***R**, each reader takes exactly one step.

Let $R^{\prime } \neq R$ be a different set of readers and let $Q^{\prime } = \{r_{1},\ldots ,r_{n}\} -R^{\prime }$. Without loss of generality, suppose there is a reader $r_{k} \in R^{\prime } - R$. In the execution from *C*_0_ determined by schedule $\mathbf {Q^{\prime }}s\mathbf {R^{\prime }}$, reader *r*_*k*_ takes a step after the signaller’s last step, so *r*_*k*_ must return *false* if it takes a step in configuration $C_{0} \mathbf {Q^{\prime }}s\mathbf {R^{\prime }}$. However, in the execution from *C*_0_ determined by schedule **Q***s***R**, reader *r*_*k*_ does not take a step after the signaller’s last step, so *r*_*k*_ must return *true* if it takes a step in configuration *C*_0_**Q***s***R**. By response obliviousness, this implies that $\mathsf {v}(C_{0}\mathbf {Q}s\mathbf {R}) \neq \mathsf {v}(C_{0}\mathbf {Q^{\prime }}s\mathbf {R^{\prime }})$.

Since this holds for any two different sets of readers *R* and $R^{\prime }$, the blackboard domain has size at least 2^*n*^. □

## The General Setting

Let ${\mathcal M} = \{M(R) : R \subseteq \{r_{1},\dots ,r_{n}\}\}$. Recall that $|{\mathcal M} | = n+1$. For any schedule *α*, let *M*(*α*) denote *M*(*R*), where *R* is the set of readers that take steps in *α*.

### **Lemma 3**

If the blackboard can only store a finite number of different values, then, from any configuration, it is possible to reach a configuration *D* such that, for any schedules *α* and β, there exists an (*M*(*α*) ∪ *M*(β) ∪{*s*})-only schedule *γ* such that *v*(*Dαγ*) = *v*(*D*β).

### *Proof*

Let *C*_0_ be an arbitrary configuration. Suppose that, for each configuration *C* reachable from *C*_0_, there are two schedules, *α* and β, such that, for each (*M*(*α*) ∪ *M*(β) ∪{*s*})-only schedule *γ*, *v*(*Cαγ*)≠*v*(*C*β).

We inductively define an infinite sequence of configurations *C*_*j*_, for *j* ≥ 0, such that *C*_*j*+ 1_ is reachable from *C*_*j*_. Given *C*_*j*_, which is reachable from *C*_0_, there exist two schedules, *α*_*j*+ 1_ and β_*j*+ 1_, such that *v*(*C*_*j*_*α*_*j*+ 1_*γ*)≠*v*(*C*_*j*_β_*j*+ 1_) for all (*M*(*α*_*j*+ 1_) ∪ *M*(β_*j*+ 1_) ∪{*s*})-only schedules *γ*. Let *C*_*j*+ 1_ = *C*_*j*_*α*_*j*+ 1_.

For *j* ≥ 1, let $M_{j} = M(\alpha _{j}) \cup M({\upbeta }_{j}) \in {\mathcal M}$. Since ${\mathcal M}$ is finite, there exists at least one set that is equal to *M*_*j*_ for an infinite number of *j*’s. Let $M^{\prime }$ denote the largest such set and let $J = \{ j \geq 1 : M_{j} = M^{\prime } \}$ be the set of indices of the occurrences of $M^{\prime }$. Let $k^{*} = {\min \limits } \{ k \geq 1 : M_{j} \subseteq M^{\prime } \textrm { for all } j \geq k \}$ be the first index after which no set larger than $M^{\prime }$ occurs. Note that, if *k*^∗^≤ *k* < *ℓ*, then *γ* = *α*_*k*+ 1_⋯*α*_*ℓ*− 1_β_*ℓ*_ is an $(M^{\prime } \cup \{s\})$-only schedule. Hence, if *k*,*ℓ* ∈ *J*, then *v*(*C*_*k*− 1_β_*k*_)≠*v*(*C*_*k*− 1_*α*_*k*_*γ*) = *v*(*C*_*ℓ*− 1_β_*ℓ*_). Thus {*v*(*C*_*k*− 1_β_*k*_) : *k* ≥ *k*^∗^ and *k* ∈ *J*} is an infinite set of values that can appear on the blackboard. □

For 0 ≤ *i* < *j* ≤ *n*, let *δ*(*i*,*j*) denote the schedule $r_{1} s r_{2} s {\dots } r_{i} s r_{i+1} r_{i+2}{\dots } r_{j}$. For example, *δ*(0,3) = *r*_1_*r*_2_*r*_3_ and *δ*(2,5) = *r*_1_*sr*_2_*sr*_3_*r*_4_*r*_5_. Note that *δ*(*i*,*j*) contains *i* occurrences of *s*.

### **Lemma 4**

Let *D* be a reachable configuration such that, for any schedules *α* and β, there exists an (*M*(*α*) ∪ *M*(β) ∪{*s*})-only schedule *γ* such that *v*(*Dαγ*) = *v*(*D*β). If 0 ≤ *i* < *j* ≤ *n*, $0 \leq i^{\prime } < j^{\prime } \leq n$, and either $i \neq i^{\prime }$ or $j \neq j^{\prime }$, then $\mathsf {v}(D\delta {(i,j)}) \neq \mathsf {v}(D\delta {(i^{\prime },j^{\prime })})$.

### *Proof*

First consider the case when $i \neq i^{\prime }$. Without loss of generality, suppose that $i < i^{\prime }$. The state of reader *r*_*i*+ 1_ is the same in configurations *Dδ*(*i*,*j*) and $D\delta {(i^{\prime },j^{\prime })}$. In configuration *Dδ*(*i*,*j*), if *r*_*i*+ 1_ takes a step, it must return *false*, because *s* has not taken any steps since *r*_*i*+ 1_ last took a step. In configuration $D\delta {(i^{\prime },j^{\prime })}$, if *r*_*i*+ 1_ takes a step, it must return *true*, because *s* has taken $i^{\prime } - i$ steps since *r*_*i*+ 1_ last took a step. If $\mathsf {v}(D\delta {(i,j)}) = \mathsf {v}(D\delta {(i^{\prime },j^{\prime })})$, then configurations *Dδ*(*i*,*j*) and $D\delta {(i^{\prime },j^{\prime })}$ are indistinguishable to *r*_*i*+ 1_, which is impossible. Thus $\mathsf {v}(D\delta {(i,j)}) \neq \mathsf {v}(D\delta {(i^{\prime },j^{\prime })})$.

Now consider the case when $i = i^{\prime }$ and $j \neq j^{\prime }$. Without loss of generality, suppose that $j < j^{\prime }$, so $\delta {(i^{\prime },j^{\prime })} = \delta {(i,j)}r_{j+1} {\cdots } r_{j^{\prime }}$. Let *α* = *δ*(*i*,*j*)*s* and β = *δ*(*i*,*j*). By assumption, there exists an {*r*_1_,…,*r*_*j*_,*s*}-only schedule *γ* such that *v*(*Dδ*(*i*,*j*)*sγ*) = *v*(*Dδ*(*i*,*j*)). To obtain a contradiction, suppose that $\mathsf {v}(D\delta {(i^{\prime },j^{\prime })}) = \mathsf {v}(D\delta {(i,j)})$. Configurations $D\delta {(i^{\prime },j^{\prime })}$ and *Dδ*(*i*,*j*) are indistinguishable to *r*_1_,…,*r*_*j*_, and *s*, since the signaller and these readers take no steps in $r_{j+1} {\cdots } r_{j^{\prime }}$. Then $ \mathsf {v}(D\delta {(i^{\prime },j^{\prime })}s\gamma ) = \mathsf {v} (D\delta {(i,j)}s\gamma ) = \mathsf {v}(D\delta {(i,j)}) = \mathsf {v}(D\delta {(i^{\prime },j^{\prime })})$. Since *r*_*j*+ 1_ does not appear in *sγ*, configurations $D\delta {(i^{\prime },j^{\prime })}s\gamma $ and $D\delta {(i^{\prime },j^{\prime })}$ are indistinguishable to *r*_*j*+ 1_. Note that *r*_*j*+ 1_ must return *false* if it takes a step in configuration $D\delta {(i^{\prime },j^{\prime })}$, because *s* has not taken any steps since *r*_*j*+ 1_ last took a step. However, *r*_*j*+ 1_ must return *true* if it takes a step in configuration $D\delta {(i^{\prime },j^{\prime })}s\gamma $, because *s* has taken a step since *r*_*j*+ 1_ last took a step. This is impossible, because $D\delta {(i^{\prime },j^{\prime })}s\gamma $ and $D\delta {(i^{\prime },j^{\prime })}$ are indistinguishable to *r*_*j*+ 1_. □

Using this lemma, we obtain Theorem 1.

### **Theorem 1**

In any algorithm for the signal detection problem, the blackboard has domain size at least *n*(*n* + 1)/2.

### *Proof*

Consider any algorithm for signal detection in which the blackboard stores a finite number of different values. By Lemma 3, there is a reachable configuration *D* such that, for any schedules *α* and β, there exists an (*M*(*α*) ∪ *M*(β) ∪{*s*})-only schedule *γ* such that *v*(*Dαγ*) = *v*(*D*β). By Lemma 4, for all different choices of 0 ≤ *i* < *j* ≤ *n*, the value of the blackboard in configuration *Dδ*(*i*,*j*) is different. There are *n*(*n* + 1)/2 ∈Ω(*n*^2^) such choices. □

## Two Readers

In this section, we examine the special case when there are exactly two readers. The simple signal detection algorithm presented in Section 1.2 with one bit for each reader has a blackboard domain of size 2^2^ = 4. First, we prove that this is optimal.

To prove our conjecture that 2^*n*^ blackboard values are necessary for *n* readers, one approach would be to show the existence of a configuration from which *P*-only executions lead to different blackboard configurations for different subsets *P* of the readers. When *n* = 2, we show that such a configuration does not always exist. In particular, in Section 7.2, we present an algorithm for two readers, *r*_1_ and *r*_2_, with the property that, from every reachable configuration *C*, any reader-only execution leads to at most three different blackboard values. Thus, in order to show that four different blackboard values can be reached from some reachable configuration *C*, the signaller must also take steps, as is the case in our lower bound in Section 7.1. We conjecture that there are similar counterexample algorithms for *n* > 2.

### A Tight Lower Bound

We begin with a straightforward observation about when two configurations have the same blackboard value.

#### **Observation 7**

For any configuration *C* and any schedule *α* such that *v*(*C*) = *v*(*Cα*), if β is a schedule consisting of processes that don’t take steps in *α*, then *v*(*C*β) = *v*(*Cα*β).

The proof follows from the fact that configurations *C* and *Cα* are indistinguishable to the processes that take steps in β.

In some situations, it is also easy to prove that two configurations have different blackboard values.

#### **Lemma 5**

If *C* is a reachable configuration, *ρ* is a schedule that contains reader *r* but not the signaller *s*, *σ* is a schedule that contains *s* but not *r*, and *γ* is a schedule that contains neither *s* nor *r*, then *v*(*Cρσ*)≠*v*(*Cργ*).

#### *Proof*

If reader *r* takes a step in configuration *Cρσ*, it must return *true*, because *r*’s previous step occurred during *ρ* and *s* took a step during *σ*. However, if *r* takes a step in configuration *Cργ*, it must return *false*, because *s* takes no steps during *ργ*.

Since *r* takes no steps in *σ* or *γ*, the state of *r* in configurations *Cρσ* and *Cργ* must be the same. Now suppose that *v*(*Cρσ*) = *v*(*Cργ*). Then configurations *Cρσ* and *Cργ* are indistinguishable to *r*, so it must return the same value if it takes a step in *Cρσ* and *Cργ*. This is a contradiction. □

#### **Corollary 1**

If *C* is a reachable configuration, *ρ* is a schedule that contains reader *r* but not the signaller *s*, and *σ* is a schedule that contains *s* but not *r*, then *v*(*Cρσ*)≠*v*(*Cρ*).

These results will be used repeatedly to obtain a tight lower bound when there are two readers.

#### **Theorem 8**

Any algorithm for signal detection among *n* = 2 readers requires blackboard domain size at least 4.

#### *Proof*

Consider any algorithm, let *C*_0_ be its initial configuration, and let *D* = *C*_0_*r*_1_*r*_2_. Since the blackboard domain size is finite, there are two integers *k*,*ℓ* ≥ 1, such that *v*(*Ds*^*k*^) = *v*(*Ds*^*k*+*ℓ*^). We first show that *v*(*Ds*^*k*^*r*_1_)≠*v*(*Ds*^*k*^)≠*v*(*Ds*^*k*^*r*_2_). To obtain a contradiction, suppose that *v*(*Ds*^*k*^) = *v*(*Ds*^*k*^*r*_*i*_) for some *i* ∈{1,2}. Then *v*(*Ds*^*k*+*ℓ*^) = *v*(*Ds*^*k*^*r*_*i*_*s*^*ℓ*^) by Observation 7 with *C* = *Ds*^*k*^, *α* = *r*_*i*_ and β = *s*^*ℓ*^. Hence *v*(*Ds*^*k*^*r*_*i*_) = *v*(*Ds*^*k*^) = *v*(*Ds*^*k*+*ℓ*^) = *v*(*Ds*^*k*^*r*_*i*_*s*^*ℓ*^). But *v*(*Ds*^*k*^*r*_*i*_*s*^*ℓ*^)≠*v*(*Ds*^*k*^*r*_*i*_) by Corollary 1 with *C* = *Ds*^*k*^, *r* = *r*_*i*_, *ρ* = *r*_*i*_, and *σ* = *s*^*ℓ*^. This is a contradiction. Therefore *v*(*Ds*^*k*^)≠*v*(*Ds*^*k*^*r*_1_),*v*(*Ds*^*k*^*r*_2_).

Applying Corollary 1 with *C* = *C*_0_, *r* = *r*_2_, *ρ* = *r*_1_*r*_2_, and *σ* = *s*^*k*^*r*_1_ gives *v*(*Ds*^*k*^*r*_1_) = *v*(*C*_0_*r*_1_*r*_2_*s*^*k*^*r*_1_)≠*v*(*C*_0_*r*_1_*r*_2_) = *v*(*D*). Similarly, *v*(*Ds*^*k*^*r*_2_)≠*v*(*D*). Applying Corollary 1 with *C* = *C*_0_, *r* = *r*_1_, *ρ* = *r*_1_*r*_2_, and *σ* = *s*^*k*^, gives *v*(*Ds*^*k*^) = *v*(*C*_0_*r*_1_*r*_2_*s*^*k*^)≠*v*(*C*_0_*r*_1_*r*_2_) = *v*(*D*). If *v*(*Ds*^*k*^*r*_1_)≠*v*(*Ds*^*k*^*r*_2_), then *v*(*D*), *v*(*Ds*^*k*^), *v*(*Ds*^*k*^*r*_1_), and *v*(*Ds*^*k*^*r*_2_) are all distinct, so suppose that *v*(*Ds*^*k*^*r*_1_) = *v*(*Ds*^*k*^*r*_2_).

Applying Corollary 1 with *C* = *C*_0_, *r* = *r*_2_, *ρ* = *r*_1_*r*_2_, and *σ* = *s*^*k*^*r*_1_*s* gives *v*(*Ds*^*k*^*r*_1_*s*) = *v*(*C*_0_*r*_1_*r*_2_*s*^*k*^*r*_1_*s*)≠*v*(*C*_0_*r*_1_*r*_2_) = *v*(*D*). Applying Corollary 1 with *C* = *Ds*^*k*^, *r* = *r*_1_, *ρ* = *r*_1_, and *σ* = *s* gives *v*(*Ds*^*k*^*r*_1_*s*)≠*v*(*Ds*^*k*^*r*_1_). If *v*(*Ds*^*k*^*r*_1_*s*)≠*v*(*Ds*^*k*^), then *v*(*D*), *v*(*Ds*^*k*^), *v*(*Ds*^*k*^*r*_1_), and *v*(*Ds*^*k*^*r*_1_*s*) are all distinct, so suppose that *v*(*Ds*^*k*^*r*_1_*s*) = *v*(*Ds*^*k*^).

By Observation 7 with *C* = *Ds*^*k*^, *α* = *r*_1_*s* and β = *r*_2_, it follows that *v*(*Ds*^*k*^*r*_1_*sr*_2_) = *v*(*Ds*^*k*^*r*_2_), so *v*(*Ds*^*k*^*r*_1_*sr*_2_) = *v*(*Ds*^*k*^*r*_1_). Applying Corollary 1 with *C* = *Ds*^*k*^, *r* = *r*_1_, *ρ* = *r*_1_, and *σ* = *sr*_2_ gives *v*(*Ds*^*k*^*r*_1_*sr*_2_)≠*v*(*Ds*^*k*^*r*_1_). This is a contradiction. Therefore, either *v*(*Ds*^*k*^*r*_1_)≠*v*(*Ds*^*k*^*r*_2_) or *v*(*Ds*^*k*^*r*_1_*s*)≠*v*(*Ds*^*k*^) and, hence, the domain size is at least 4. □

### An Algorithm for Two Readers Based on a Bounded Sequential Timestamp System

A *bounded sequential timestamp system* for a set of processes consists of a finite set of labels, *L*, and a *dominance* relation ≺ between every two different labels, i.e. if *ℓ*_1_ and *ℓ*_2_ are two different labels then exactly one of *ℓ*_1_ ≺ *ℓ*_2_ and *ℓ*_2_ ≺ *ℓ*_1_ is true. At every configuration, each process has a different label. There is one atomic operation, getTimestamp(), which may change the label of the process that called it and which ensures that its label dominates the labels of all other processes. A solution to the signal detection problem can be obtained from a bounded sequential timestamp system by using the blackboard to store the timestamps of the signaller and each reader. When either a reader or the signaller takes a step, it performs getTimestamp() and updates its timestamp stored on the blackboard, if it has changed. A reader returns *true* if and only if its timestamp was smaller (with respect to ≺) than the timestamp of the signaller before it was updated. The domain size of the blackboard is |*L*|^*n*+ 1^ in this algorithm, where *n* is the number of readers. Note that this algorithm provides a lot more information than is required by the signal detection problem. Specifically, each reader can also determine which of the other readers have taken steps since it last took a step.

Israeli and Li [5] gave a construction of a bounded sequential timestamp system for *n* + 1 processes with a label set of size 3^*n*^ and proved that, for any *𝜖* > 0, there exists a bounded sequential timestamp system for *n* + 1 processes whose label set has size (2 + *𝜖*)^*n*+ 1^, for *n* sufficiently large. They also proved a lower bound of 2^*n*+ 1^ − 1 for the size of the label set of any bounded sequential timestamp system for *n* + 1 processes.

The algorithm below is based on the bounded timestamp system of Israeli and Li for three processes with 3^2^ = 9 labels, but it stores only two labels on the blackboard: the signaller’s label and the larger (with respect to ≺) of the two readers’ labels. This results in a blackboard with domain size 81, which is much larger than the number of labels used in the optimal algorithm. However, this algorithm has the property that, starting from any reachable configuration, at most three different blackboard configurations can be reached by executions in which only readers take steps.

Let *B* = {0,1,2}. The set of labels is *B*^2^ = *B* × *B*. We define the dominance relation ≺ as follows: For (*a*_1_,*a*_0_),(*b*_1_,*b*_0_) ∈ *B*^2^, (*a*_1_,*a*_0_) ≺ (*b*_1_,*b*_0_) if and only if

1. *b*_1_ ≡ *a*_1_ + 1 (mod 3) or
2. *b*_1_ = *a*_1_ and *b*_0_ ≡ *a*_0_ + 1 (mod 3).

Note that, for any two distinct labels *a*,*b* ∈ *B*^2^, either *a* ≺ *b* or *b* ≺ *a*. Israeli and Li view the set of all labels with the same first component as being the labels of a separate copy of a bounded sequential timestamp for 2 processes (which uses labels in *B*).

The algorithm maintains the following *label invariants*: In each reachable configuration, each process *p* ∈{*s*,*r*_0_,*r*_1_} has a different label $\ell (p)=\left (\ell _{1}(p),\ell _{0}(p)\right )\in B^{2}$. In addition, each reader has a label from a different copy (i.e. *ℓ*_1_(*r*_0_)≠*ℓ*_1_(*r*_1_)) and the signaller has a label from one of these copies (i.e. *ℓ*_1_(*s*) ∈{*ℓ*_1_(*r*_0_),*ℓ*_1_(*r*_1_)}). In the initial configuration,

$$ \ell(r_{0})=(0,0),\ \ell(r_{1})=(1,0),\ \text{and}\ \ell(s)=(1,1), $$

so the label invariants are satisfied.

In each reachable configuration, the readers have different labels, so one of the reader’s labels dominates the other reader’s label. We call the reader with this label the *dominating reader*.

B $$ \begin{array}{@{}rcl@{}} &&\text{The blackboard stores the pair}~ \left( \ell(s),\ell(r_{d})\right),\\ && \text{where} d\in\{0,1\} \text{and} \ell(r_{1-d})\prec\ell(r_{d}). \end{array} $$

In other words, the signaller’s label as well as the dominating reader’s label are stored on the blackboard. Thus, initially, the blackboard contains ((1,1),(1,0)).

When a process takes a step, it applies the rules described below, which might change its label. We prove that the label invariants remain satisfied. If its label is changed, the process updates the blackboard so that (B) remains true. We will show that, in addition,

S $$ \begin{array}{@{}rcl@{}} && \text{immediately after each step by } s, \text{ its label } \ell(s) \text{ dominates both } \ell(r_{0}) \text{ and } \ell(r_{1}) \text{ and}\\ && \text{immediately after each step by a reader } r_{i}, \text{ its label } \ell(r_{i}) \text{ dominates } \ell(s). \end{array} $$

Therefore, a reader can easily provide the proper response when it takes a step: If a reader’s label dominates *ℓ*(*s*) at the beginning of its step, then the reader returns *false*. Otherwise it returns *true*.

Consider any configuration that satisfies (B) and the label invariants. Let *d* ∈{0,1} be such that *ℓ*(*r*_1−*d*_) ≺ *ℓ*(*r*_*d*_). Since *ℓ*_1_(*r*_1−*d*_)≠*ℓ*_1_(*r*_*d*_), it follows that *ℓ*_1_(*r*_*d*_) ≡ *ℓ*_1_(*r*_1−*d*_) + 1 (mod 3).

*The Signaller’s Step.*: When the signaller *s* takes a step, it compares its label with *ℓ*(*r*_*d*_), which is stored on the blackboard. If *ℓ*(*r*_*d*_) ≺ *ℓ*(*s*), then *s* retains its old label and does not update the blackboard, so (B) and the label invariants remain satisfied. In this case, *ℓ*_1_(*s*)≠*ℓ*_1_(*r*_1−*d*_), so, by the label invariants, *ℓ*_1_(*s*) = *ℓ*_1_(*r*_*d*_) and, hence, *ℓ*(*r*_1−*d*_) ≺ *ℓ*(*s*). Thus, (S) is true.
  Now consider the case when *ℓ*(*s*) ≺ *ℓ*(*r*_*d*_). The new label of *s* is
  $$ \begin{array}{@{}rcl@{}} \ell^{\prime}(s)=(\ell_1(r_d),(\ell_0(r_d)+1)\text{mod} 3). \end{array} $$
  By definition, $\ell (r_{d})\prec \ell ^{\prime }(s)$. Since $\ell _{1}^{\prime }(s) = \ell _{1}(r_{d}) \equiv \ell _{1}(r_{1-d}) +1 ~(\text {mod}~ 3)$, we have $\ell (r_{1-d}) \prec \ell ^{\prime }(s)$. Thus (S) is satisfied and the labels of all three processes are different. By definition, $\ell ^{\prime }_{1}(s) = \ell _{1}(r_{d}) \in \{\ell _{1}(r_{0}), \ell _{1}(r_{1})\}$. Since the labels of *r*_0_ and *r*_1_ do not change, *ℓ*_1_(*r*_0_)≠*ℓ*_1_(*r*_1_). Hence, the label invariants remain satisfied. After determining its new label $\ell ^{\prime }(s)$, the signaller updates the first component of the pair stored on the blackboard with $\ell ^{\prime }(s)$, so (B) remains satisfied.
*The Reader’s Step.*: When reader *r*_*i*_, *i* ∈{0,1}, takes a step, it compares its label with *ℓ*(*s*), which is stored on the blackboard. If *ℓ*(*s*) ≺ *ℓ*(*r*_*i*_), then *r*_*i*_ does not change its label and (S) is true. In this case, none of the label invariants are affected, the reader does not change the blackboard, and (B) remains satisfied.

So suppose that *ℓ*(*r*_*i*_) ≺ *ℓ*(*s*). Then *r*_*i*_’s new label is

$$ \ell^{\prime}(r_{i})= \begin{cases} \left( \ell_{1}(s),(\ell_{0}(s)+1)\text{mod} 3\right) & \text{if } \ell_{1}(r_{i})=\ell_{1}(s)\\ \left( (\ell_{1}(s)+1)\text{mod} 3,0\right) & \text{otherwise.} \end{cases} $$

In both cases, $\ell (s)\prec \ell ^{\prime }(r_{i})$, so (S) is satisfied and $\ell ^{\prime }(r_{i}) \neq \ell (s)$. If *ℓ*_1_(*r*_*i*_) = *ℓ*_1_(*s*), then, by construction, $\ell _{1}^{\prime }(r_{i})=\ell _{1}(s)=\ell _{1}(r_{i})$. By the label invariants, *ℓ*_1_(*r*_*i*_)≠*ℓ*_1_(*r*_1−*i*_), so $\ell _{1}^{\prime }(r_{i}) \neq \ell _{1}(r_{1-i})$. If *ℓ*_1_(*r*_*i*_)≠*ℓ*_1_(*s*), then, since *ℓ*_1_(*s*) ∈{*ℓ*_1_(*r*_0_),*ℓ*_1_(*r*_1_)}, we have *ℓ*_1_(*s*) = *ℓ*_1_(*r*_1−*i*_). By construction, $\ell _{1}^{\prime }(r_{i})\neq \ell _{1}(s)$, so $\ell _{1}^{\prime }(r_{i}) \neq \ell _{1}(r_{1-i})$. In both cases, $\ell ^{\prime }(r_{i}) \neq \ell (r_{1-i})$. Since *ℓ*(*r*_1−*i*_)≠*ℓ*(*s*), the labels of all three processes are different. Hence, the label invariants remain satisfied.

If *ℓ*_1_(*r*_*i*_) = *ℓ*_1_(*s*) and *d* = 1 − *i* (i.e., the second label on the blackboard is not equal to *ℓ*(*r*_*i*_)), then *r*_*i*_ does not change the blackboard. In this case, by (B), *ℓ*(*r*_*i*_) ≺ *ℓ*(*r*_1−*i*_) and, by the label invariants, *ℓ*_1_(*r*_*i*_)≠*ℓ*_1_(*r*_1−*i*_). Therefore, it follows that *ℓ*_1_(*r*_1−*i*_) ≡ (*ℓ*_1_(*r*_*i*_) + 1) (mod 3). By construction, $\ell ^{\prime }_{1}(r_{i}) = \ell _{1}(s)$, so $\ell _{1}(r_{1-i}) \equiv (\ell ^{\prime }_{1}(r_{i}) +1) ~(\text {mod}~ 3)$ and, hence, $\ell ^{\prime }(r_{i}) \prec \ell (r_{1-i})$. Thus (B) remains satisfied.

If *ℓ*_1_(*r*_*i*_) = *ℓ*_1_(*s*) and *d* = *i*, then *r*_*i*_ changes the second component of the blackboard to $\ell ^{\prime }(r_{i}) = \left (\ell _{1}(s),(\ell _{0}(s)+1)\text {mod} 3\right )$. Since *ℓ*(*r*_1−*i*_) ≺ *ℓ*(*r*_*i*_) and, by the label invariants, *ℓ*_1_(*r*_1−*i*_)≠*ℓ*_1_(*r*_*i*_), it follows that $\ell ^{\prime }_{1}(r_{i}) \equiv \ell _{1}(r_{1-i}) +1 ~(\text {mod}~ 3)$. Hence $\ell (r_{1-i}) \prec \ell ^{\prime }(r_{i})$ and (B) remains satisfied.

The last case is when *ℓ*_1_(*r*_*i*_)≠*ℓ*_1_(*s*). Then *r*_*i*_ changes the second component of the blackboard to $\ell ^{\prime }(r_{i}) = \left ((\ell _{1}(s)+1)\text {mod} 3,0\right )$. By the label invariants, *ℓ*_1_(*s*) = *ℓ*_1_(*r*_1−*i*_), so $\ell ^{\prime }_{1}(r_{i}) = (\ell _{1}(r_{1-i})+1) \text {mod} 3$. Hence $\ell (r_{1-i}) \prec \ell ^{\prime }(r_{i})$ and (B) remains satisfied.

#### **Lemma 6**

For any reachable configuration *C*,

$$ \begin{array}{@{}rcl@{}} \left|\left\{\mathsf{v}(C\alpha): \alpha\in\{r_0,r_1\}^\ast \right\}\right|\leq 3. \end{array} $$

#### *Proof*

Without loss of generality, we may assume that either *C* is the initial configuration or the last step in the execution leading to *C* was taken by the signaller. Let $\ell ^{\ast }(p)=\left (\ell _{1}^{\ast }(p),\ell _{0}^{\ast }(p)\right )$ denote the label of process *p* ∈{*s*,*r*_0_,*r*_1_} in configuration *C*. By (S) and the definition of the initial configuration, the label of the signaller, *ℓ*^∗^(*s*), dominates the labels, *ℓ*^∗^(*r*_0_) and *ℓ*^∗^(*r*_1_), of the readers. By the label invariants, there is an index *d* ∈{0,1} such that $\ell _{1}^{\ast }(s)=\ell _{1}^{\ast }(r_{d})\neq \ell _{1}^{\ast }(r_{1-d})$. Since *ℓ*^∗^(*r*_1−*d*_) is dominated by *ℓ*^∗^(*s*), it is also dominated by *ℓ*^∗^(*r*_*d*_). Thus, by (B), $\mathsf {v}(C)=\left (\ell ^{\ast }(s),\ell ^{\ast }(r_{d})\right )$.

The signaller takes no steps in *α*, so its label remains unchanged. If *r*_*d*_ takes at least one step in *α*, its label changes to $\ell ^{\prime }(r_{d}) =\left (\ell _{1}^{\ast }(s),(\ell _{0}^{\ast }(s)+1)\text {mod} 3\right )$ and, if *r*_1−*d*_ takes at least one step in *α*, its label changes to $ \ell ^{\prime }(r_{1-d}) ={\left ((\ell _{1}^{\ast }(s)+1)\text {mod} 3,0\right )}$. By (S), after a reader has taken a step, its new label dominates *ℓ*^∗^(*s*), so its label does not change when it takes additional steps in *α*.

When a reader takes a step, either it does not change the blackboard, or it replaces the second component of the blackboard with its new label. Thus, for all $\alpha \in \{r_{0},r_{1}\}^{\ast }$,

$$ \begin{array}{@{}rcl@{}} \mathsf{v}(C\alpha)=(\ell^\ast(s), \ell), \ \text{where}\ \ell\in\left\{\ell^\ast(r_d),\ell^{\prime}(r_d),\ell^{\prime}(r_{1-d})\right\}. \end{array} $$

Hence, $\left |\left \{\mathsf {v}(C\alpha ): \alpha \in \{r_{0},r_{1}\}^{\ast }\right \}\right |\leq 3$. □

## Discussion

This paper introduces the signal detection problem, gives some simple algorithms for solving it, proves that domain size at least *n*(*n* + 1)/2 is necessary, and proves that domain size 2^*n*^ is necessary and sufficient for three restricted versions of the problem: when the processes are write oblivious, when the readers are response oblivious, or when the signaller writes a fixed sequence of values to the blackboard. We conjecture that domain size must be at least 2^*n*^ for the unrestricted version of the problem. It would also be interesting to prove this bound for two other restricted versions of the problem: when only the signaller is write oblivious or only the readers are write oblivious.

We also consider another restricted version of the problem, where each reader takes no more than *b* ≥ 2 steps. We give an algorithm with domain size (*b* − 1)*n* + 1 and prove this is optimal when *b* = 2. It remains open whether it is optimal when *b* > 2. If the signaller takes no more that *B* steps, then one of our simple algorithms uses a domain of size *B* + 1. It is unclear whether it is possible to have an algorithm for this restricted version that has a smaller domain.
